# Sodium fluoride induces splenocyte autophagy via the mammalian targets of rapamycin (mTOR) signaling pathway in growing mice

**DOI:** 10.18632/aging.101499

**Published:** 2018-07-22

**Authors:** Ping Kuang, Huidan Deng, Huan Liu, Hengmin Cui, Jing Fang, Zhicai Zuo, Junliang Deng, Yinglun Li, Xun Wang, Ling Zhao

**Affiliations:** 1 College of Veterinary Medicine, Sichuan Agricultural University, Wenjiang, Chengdu 611130, China; 2 Key Laboratory of Animal Diseases and Environmental Hazards of Sichuan Province, Sichuan Agriculture University, Wenjiang, Chengdu 611130, China; 3 Key Laboratory of Agricultural information engineering of Sichuan Province, Sichuan Agriculture University, Yaan, Sichuan 625014, China; * These authors contributed equally to this work

**Keywords:** sodium fluoride, autophagy, mTOR signaling pathway, spleen, mouse

## Abstract

Fluoride is known to impair organism’s development and function via adverse effects, and autophagy plays a regulation role in human or animal health and disease. At present, there are no reports focused on fluoride-induced autophagy in the animal and human spleen. The objective of this study was to investigate sodium fluoride (NaF)-induced splenocyte autophagy and the potential mechanism via regulation of p-mTOR in growing mice by using the methods of transmission electron microscopy (TEM), immunohistochemistry (IHC), quantitative real-time polymerase chain reaction (qRT-PCR) and western blot. A total of 240 ICR mice were equally allocated into four groups with intragastric administration of distilled water in the control group and 12, 24, 48 mg/kg NaF solution in the experimental groups for 42 days. Results revealed that NaF increased autophagosomes or autolysosomes in spleen. Simultaneously, the autophagy marker LC3 brown punctate staining was increased with NaF dosage increase. On the other hand, NaF caused inhibition of mTOR activity, which was characterized by down-regulation of PI3K, Akt and mTOR mRNA and protein expression levels. And the suppression of mTOR activity in turn resulted in the significantly increased of ULK1 and Atg13 expression levels. Concurrently, NaF increased the levels of mRNA and protein expression of autophagy markers LC3, Beclin1, Atg16L1, Atg12, Atg5 and decreased the mRNA and protein expression levels of p62. The above-mentioned findings verify that NaF induces autophagy via mTOR signaling pathway. The inhibition of mTOR activity and alteration of autophagy-related genes and proteins are the potential molecular mechanism of NaF-induced splenocyte autophagy.

## Introduction

Fluorine is one of the essential trace elements for human health, and is widely used as a cofactor in medicine, e.g., anesthetics, antibiotics, anti-cancer and anti-inflammatory agents, and psychopharmaceuticals [1,2]. However, excessive fluoride intake can cause tissue damage and lead to multiple organ dysfunction [3,4], which depends not only on the concentration and exposed duration [5], but also on the absorption capacity, age, and nutritional status of the individual [6]. It has been demonstrated that fluoride can induce skeletal and non-skeletal fluorosis [4,7–9]. We have also confirmed that sodium fluoride (NaF) inhibits cell proliferation and induces cell apoptosis in splenic lymphocytes from mice in vivo and in vitro [10–14], and causes blood immunotoxicity from mice [15]. Other studies have also shown the fluoride-caused cytotoxicity, apoptosis and DNA damage in human and animals [16–19].

Autophagy is a degradative process by which cytoplasmic constituents of cells are engulfed within a cytoplasmic vacuole and delivered to the lysosome for degradation [20]. Autophagy plays key roles in cellular homeostasis during embryonic development, postnatal cell survival, and death [21]. Moreover, autophagy can be induced by various stress stimuli, such as oxidative stress and environmental factors [22,23]. Fluoride, as an environmental and dietary factor, had been reported to induce oxidative stress [24] and endoplasmic reticulum stress [14] in the spleen. Several studies have shown that fluoride activates autophagy via diverse signaling [25–27] in different cell lines [26,28,29], and fluoride exposure is associated with autophagy and autophagy exerts its miscellaneous function to protect or impair organism [30,31]. However, the molecular mechanism of autophagy induced by fluoride in spleen is still poorly understood. Also, there are no reports focused on fluoride-induced autophagy in the animal and human spleen at present.

In this study, mice were used to explore how sodium fluoride (NaF) induced splenic autophagy. And we demonstrated that NaF changed genes and proteins expressions of autophagy markers, including Beclin1, autophagy-related protein (Atg):16-like1(Atg16L1), 12 (Atg12), 5 (Atg5), microtubuleassociatedprotein1 light chain 3 (LC3) and p62 (SQSTM1) and increased numbers of autophagosomes or autolysosomes, and LC3 brown punctate staining in spleen by transmission electron microscopy (TEM) and by immunohistochemistry (IHC), respectively.

Further, the critical molecular regulator genes, including phosphorylations of mammalian targets of rapamycin (p-mTOR), phosphorylations of unc-51 like kinase 1(p-ULK1), Atg13, phosphatidylinositol 3-kinase (PI3K) and protein kinase B, PKB (Akt) in spleen were investigated to elucidate the association between fluoride-induced autophagy and the regulation of mTOR phosphorylation. The results may provide new insights for further understanding the role of autophagy in fluoride-induced splenic damage and toxicity.

## RESULTS

### Effects of NaF on morphological changes in mice spleen

#### Detection of autophagosomes and autolysosome in spleen

**Figure 1:**
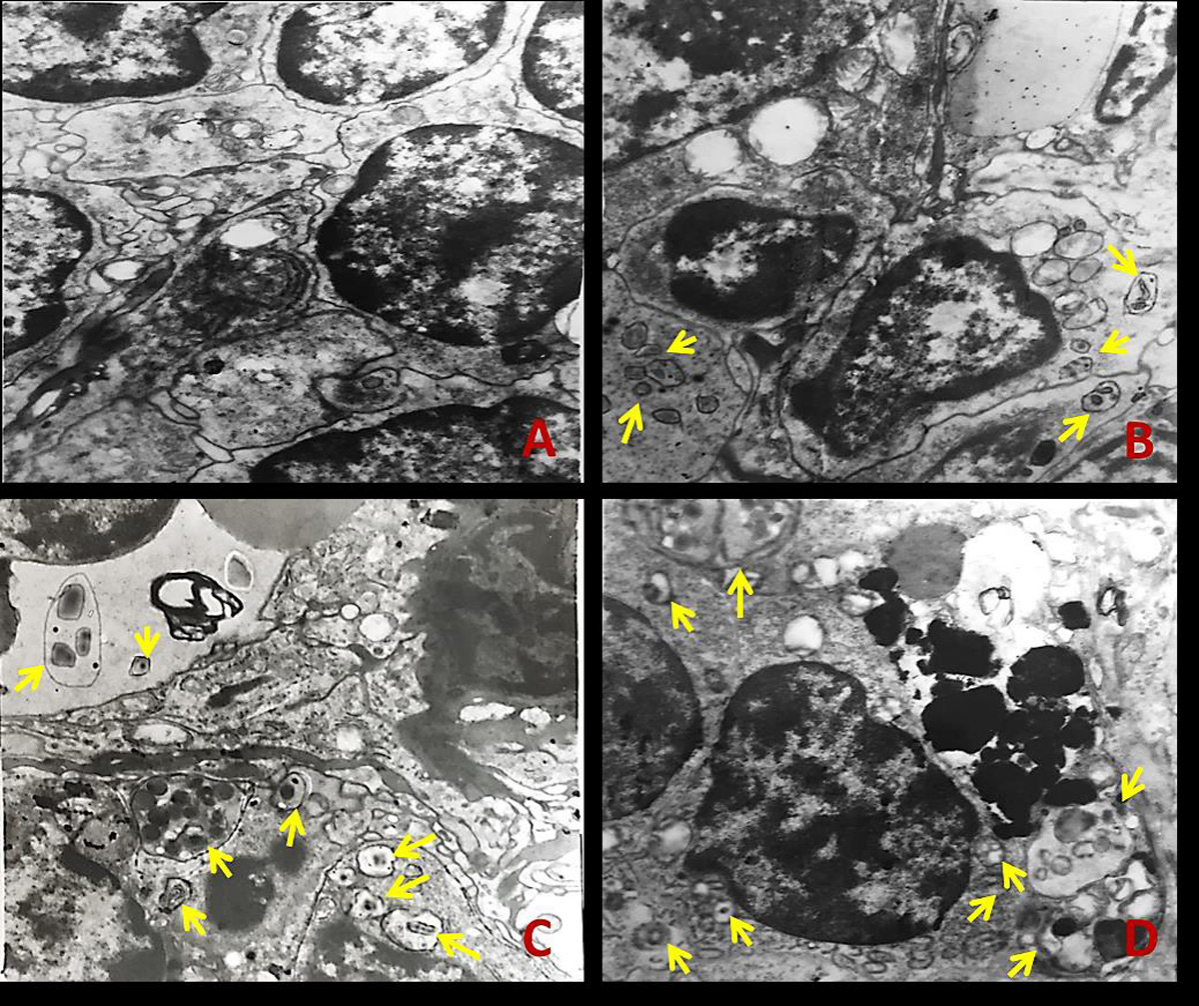
**The ultrastructure of spleen tissue by transmission electron microscopy (TEM) at 42 days of the experiment.** The yellow arrows represent autophagosome or autolysosome. (**A**) Control group; (**B**) 12 mg/kg group; (**C**) 24 mg/kg group; (**D**) 48 mg/kg group.

The splenic ultrastructure determined by TEM (Figure1) demonstrated that numbers of autophagosomes or autolysosomes were distinctly increased in NaF-treated groups when compared to the control group at 42 days of the experiment (Figure 1B-1D).

#### Detection of autophagy marker LC3 in spleen

**Figure 2:**
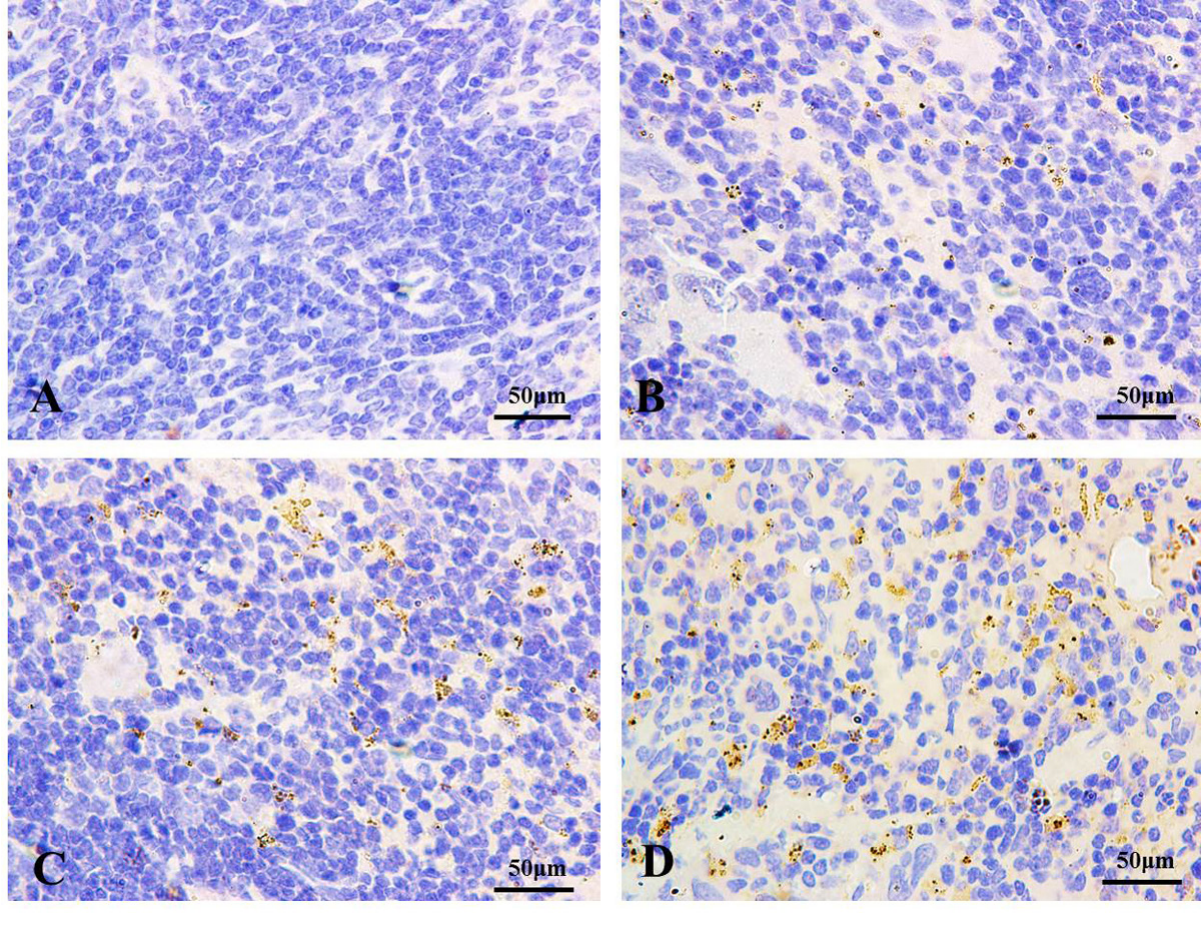
**The autophagy marker LC3 detection by immunohistochemistry (IHC) at 21 days of the experiment (100X).** The brown punctate staining represents LC3 expression. (**A**) Control group; (**B**) 12 mg/kg group; (**C**) 24 mg/kg group; (**D**) 48 mg/kg group.

**Figure 3:**
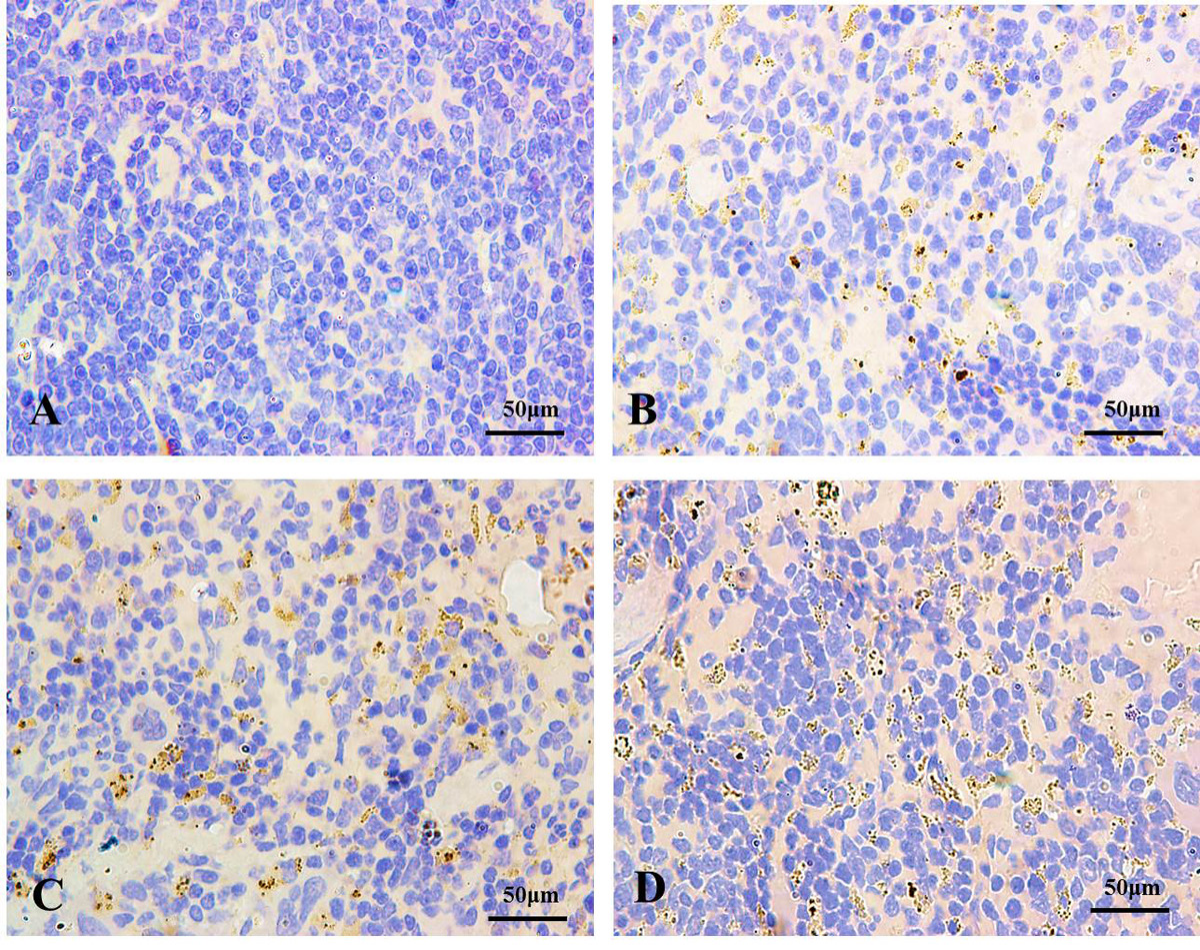
**The autophagy marker LC3 detection by immunohistochemistry (IHC) at 42 days of the experiment (100X).** The brown punctate staining represents LC3 expression. (**A**) Control group; (**B**) 12 mg/kg group; (**C**) 24 mg/kg group; (**D**) 48 mg/kg group.

Immunohistochemistry for LC3 was showed in Figures 2 and 3. In control groups, there was no punctate staining (Figures 2A and 3A). In NaF-treatment groups, the numbers of brown punctate staining were obviously increased, especially at 42 days of the experiment (Figures 2B-2D and 3B-3D).

### Effects of NaF on autophagy markers mRNA and protein expression in spleen

#### Changes of mRNA and protein expression levels of LC3, p62 and Beclin1 in spleen

**Figure 4:**
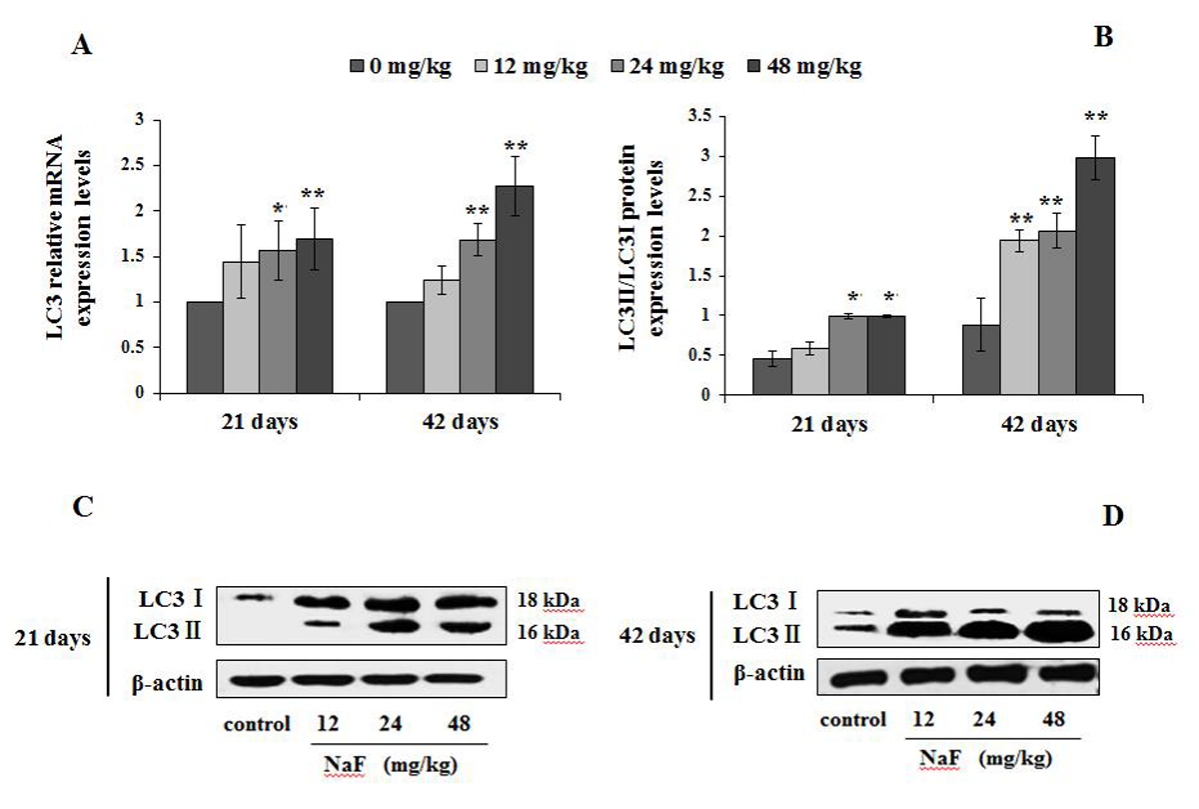
**Changes of mRNA and protein expression levels of LC3 in the spleen at 21 and 42 days of the experiment.** (**A**) The relative mRNA expression levels. (**B**) The ratio of LC3II/LC3I protein expression. (**C, D**) The western blot assay. Data are presented with the mean + standard deviation (n=8), *p < 0.05, compared with the control group; **p < 0.01, compared with the control group.

**Figure 5:**
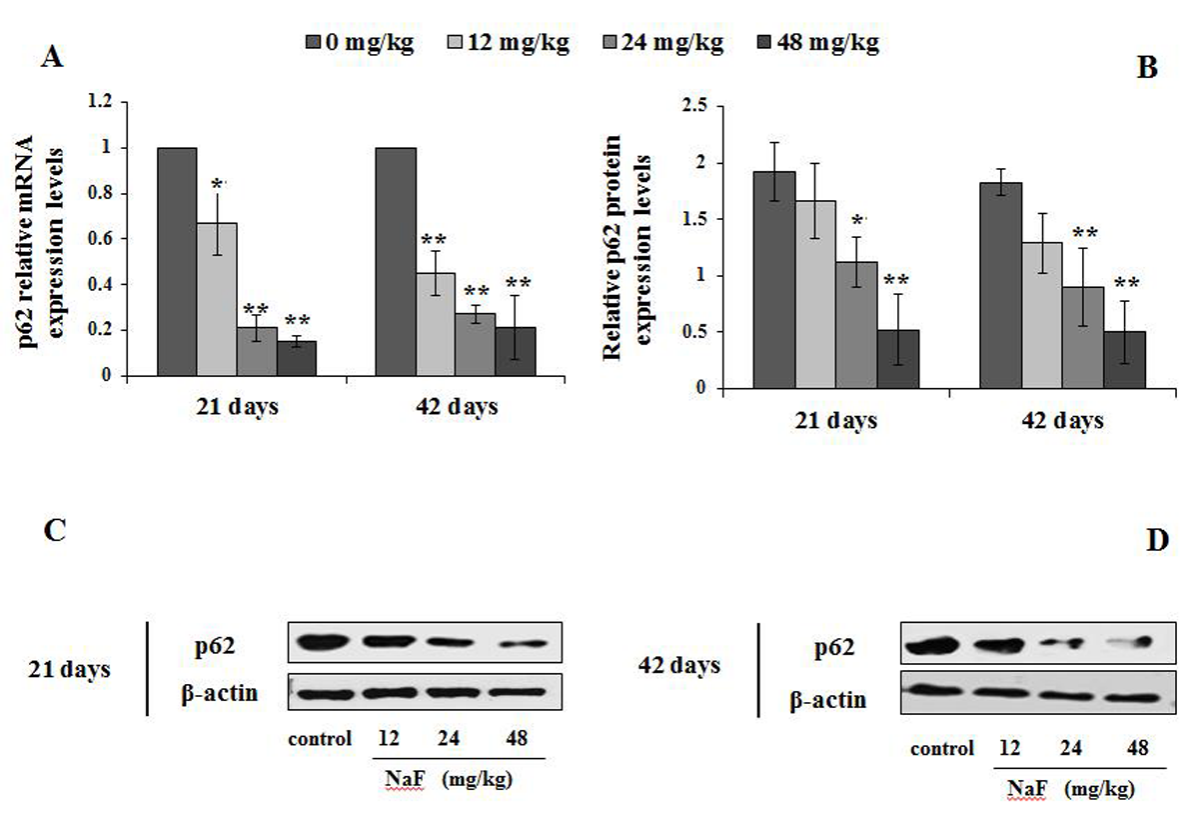
**Changes of mRNA and protein expression levels of p62 in the spleen at 21 and 42 days of the experiment.** (**A**) The relative mRNA expression levels. (**B**) The relative protein expression levels. (**C, D**) The western blot assay. Data are presented with the mean + standard deviation (n=8), *p < 0.05, compared with the control group; **p < 0.01, compared with the control group.

**Figure 6:**
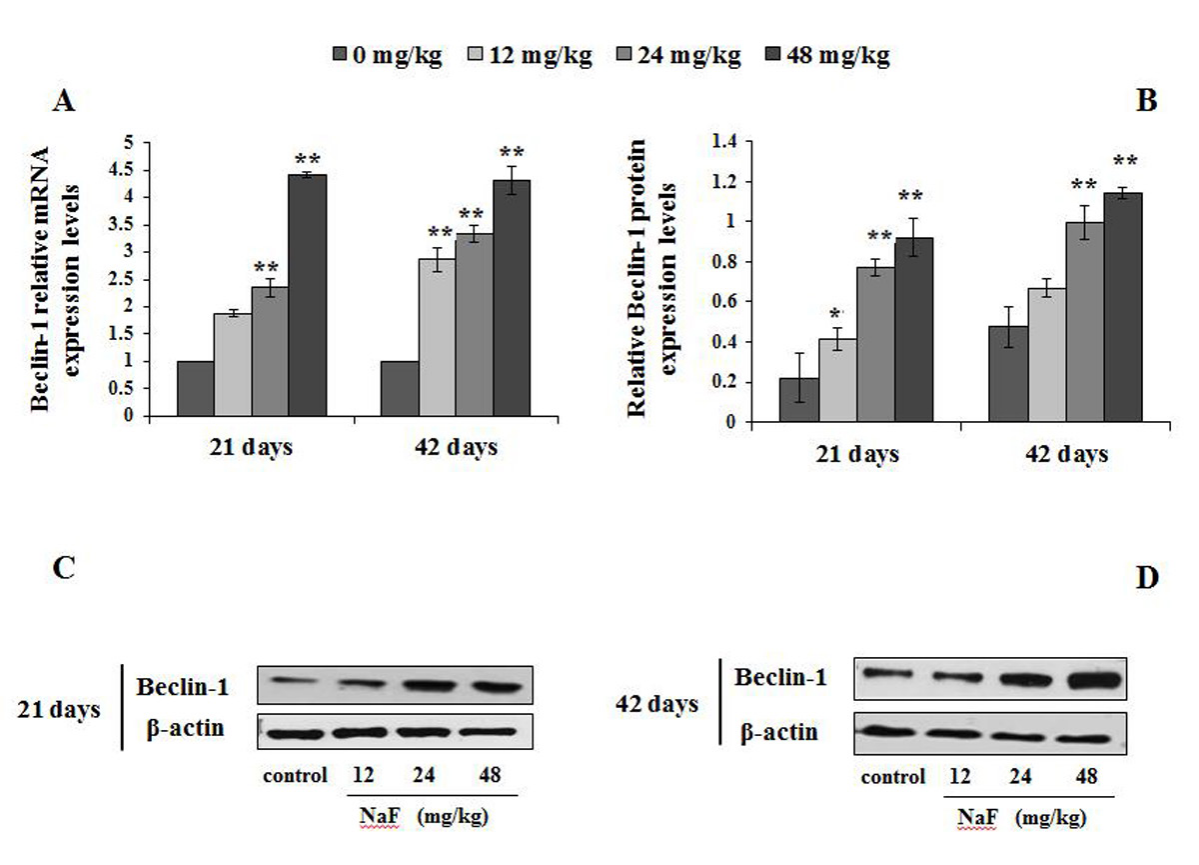
**Changes of mRNA and protein expression levels of Beclin-1 in the spleen at 21 and 42 days of the experiment.** (**A**) The relative mRNA expression levels. (**B**) The relative protein expression levels. (**C, D**) The western blot assay. Data are presented with the mean + standard deviation (n=8), *p < 0.05, compared with the control group; **p < 0.01, compared with the control group.

The mRNA and protein levels of autophagy markers, e.g LC3, Beclin1, and p62 were examined by qRT-PCR and WB (Figures 4-6).

A significantly increase in the LC3 mRNA expression levels were noted in splenocytes of the 24 and 48 mg/kg groups (P<0.05 or p<0.01) at 21 and 42 days of the experiment (Figure 4A). The ratio of LC3II/LC3I protein expression (Figure 4B-4D) was increased significantly in 24 and 48 mg/kg groups (P<0.05) at 21 days of age, and in the three NaF-treated groups (P<0.01) at 42 days of the experiment when compared to control group.

The p62 mRNA expression levels were significantly declined (P<0.05 or P<0.01) in the 12, 24 and 48 mg/kg groups (Figure 5A), and its protein expression levels were significantly decreased (P<0.05 or P<0.01) in 24 and 48 mg/kg groups (Figure 5B-5D) at 21 days and 42 days of the experiment when compared to the control group.

Figure 6A-6D showed the mRNA and protein expression levels of Beclin1. From Figure 6A, the Beclin1 mRNA expression levels were higher (P<0.05 or P<0.01) in the 12, 24 and 48 mg/kg groups than those in the control group from 21 to 42 days of the experiment. Similarly, Beclin1 protein expression levels were markedly increased (P<0.05 or P<0.01) in the 12, 24 and 48 mg/kg groups (Figure 6B-6D).

#### Changes of mRNA and protein expression levels of Atg12, Atg5 and Atg16L1 in spleen

**Figure 7:**
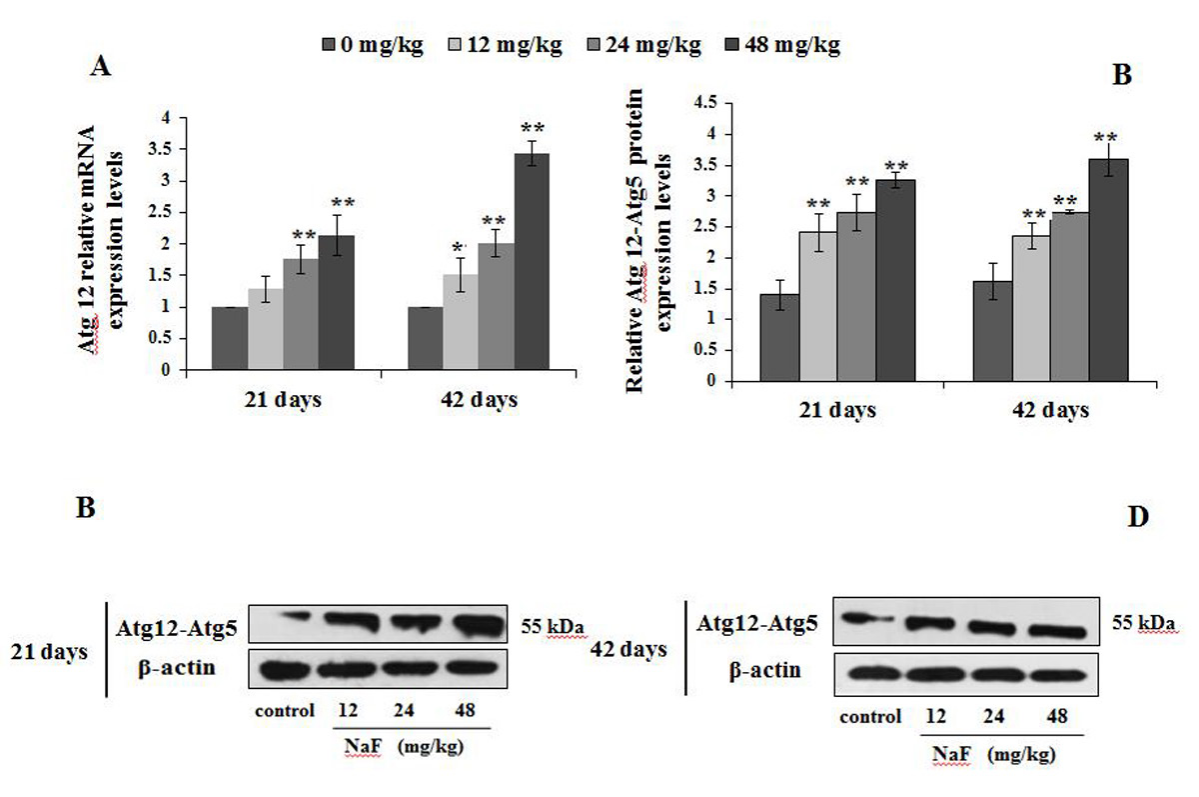
**Changes of mRNA and protein expression levels of Atg12 in the spleen at 21 and 42 days of the experiment.** (**A**) The relative mRNA expression levels. (**B**) The relative protein expression levels of Atg12-Atg5. (**C, D**) The western blot assay of Atg12-Atg5. Data are presented with the mean + standard deviation (n=8), *p < 0.05, compared with the control group; **p < 0.01, compared with the control group.

**Figure 8:**
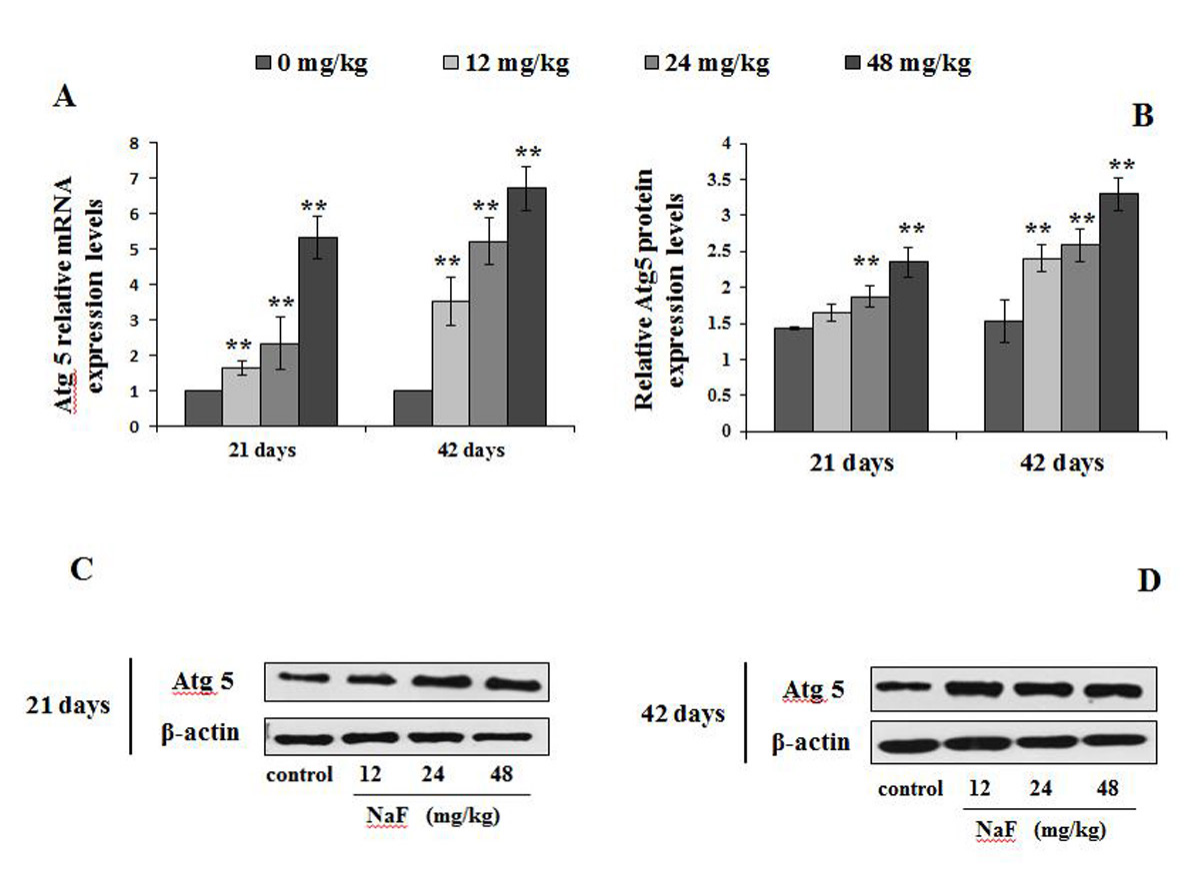
**Changes of mRNA and protein expression levels of Atg5 in the spleen at 21 and 42 days of the experiment.** (**A**) The relative mRNA expression levels. (**B**) The relative protein expression levels. (**C, D**) The western blot assay. Data are presented with the mean + standard deviation (n=8), *p < 0.05, compared with the control group; **p < 0.01, compared with the control group.

**Figure 9:**
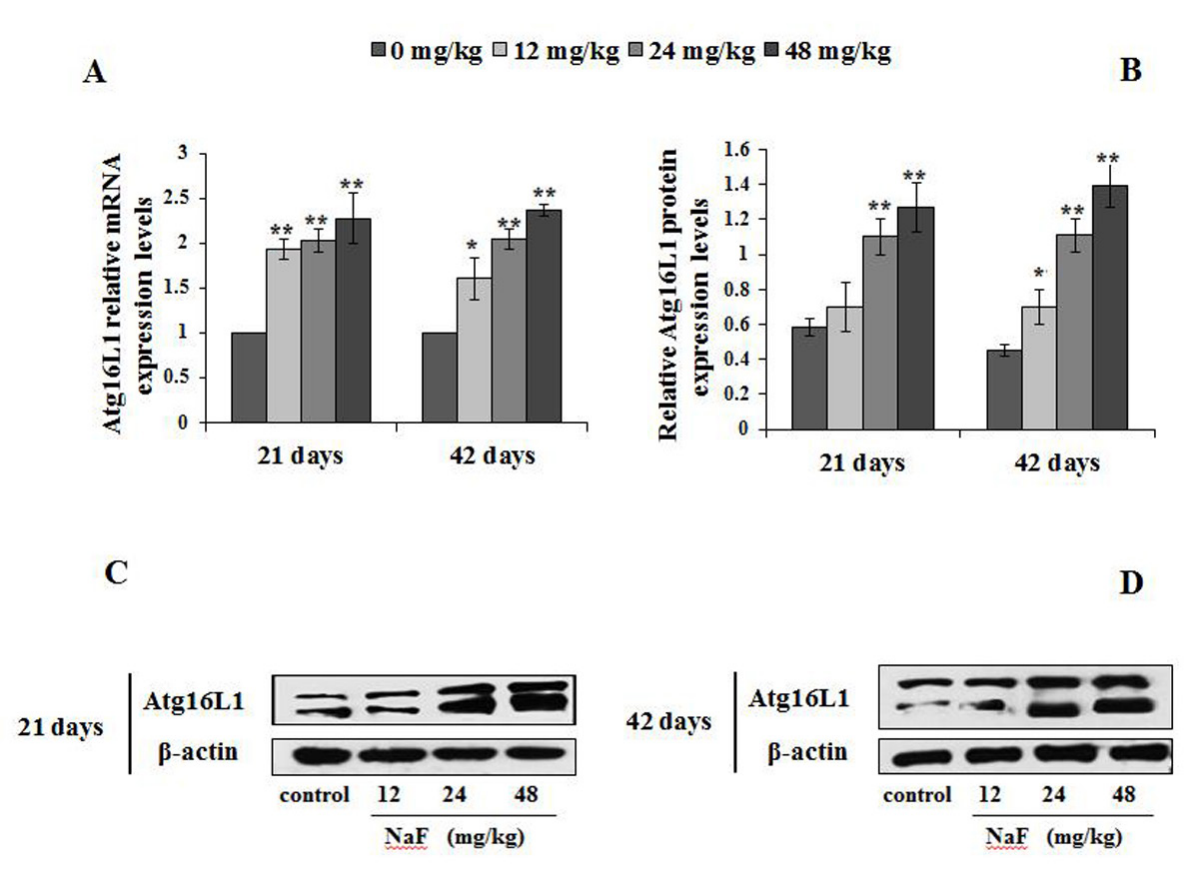
**Changes of mRNA and protein expression levels of Atg16L1 in the spleen at 21 and 42 days of the experiment.** (**A**) The relative mRNA expression levels. (**B**) The relative protein expression levels. (**C, D**) The western blot assay. Data are presented with the mean + standard deviation (n=8), *p < 0.05, compared with the control group; **p < 0.01, compared with the control group.

Next, we analyzed other autophagy markers including Atg12, Atg5 and Atg16L1, which also serve as a critical node in the autophagy signaling pathway (Figures 7-9).

We observed higher mRNA expression levels of Atg12 and Atg5 in the 12, 24 and 48 mg/kg groups at We observed higher mRNA expression levels of Atg12 and Atg5 in 21 and 42 days of the experiment than those in the control group (P<0.01 or P<0.05; Figures 7A and 8A). Additionally, Atg12-Atg5 complex protein expression levels in the three NaF-treated groups were significantly increased (P<0.01) at both 21 and 42 days of age in comparison to the control group (Figure 7B-7D). Similarly, NaF treatment markedly increased (P<0.01) the Atg5 protein expression levels in the 24 and 48 mg/kg at 21 days of the experiment, and in the 12, 24 and 48 mg/kg groups at 42 days of the experiment when compared to the control group (Figure 8B-8D).

In Figure 9A, the Atg16L1mRNA expression levels were significantly increased (P<0.05 or P<0.01) in the 12, 24 and 48 mg/kg groups from 21 to 42 days of the experiment when compared to the control group. Moreover, Atg16L1 protein expression levels were higher (P<0.01) in the 24 and 48 mg/kg groups at 21 days of the experiment, and in the 12, 24 and 48 mg/kg groups at 42 day of the experiment than those in the control group (Figure 9B-9D).

### Effects of NaF on autophagy pathway regulators mRNA and protein expression in spleen

#### Changes of mRNA and protein expression levels of phosphorylated mTOR in spleen

**Figure 10:**
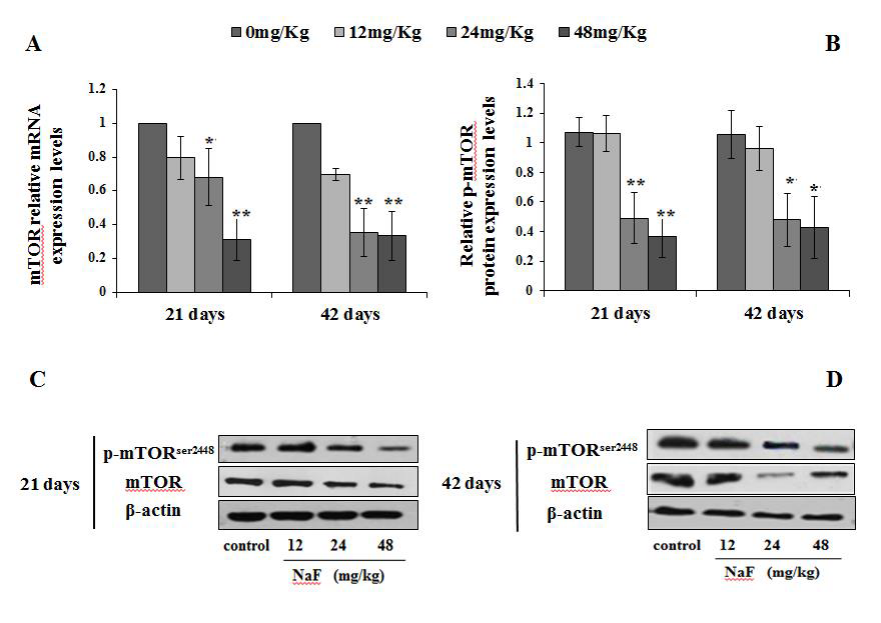
**Changes of mRNA and protein expression levels of p-mTOR in the spleen at 21 and 42 days of the experiment.** (**A**) The relative mRNA expression levels. (**B**) The relative protein expression levels. (**C, D**) The western blot assay. Data are presented with the mean + standard deviation (n=8), *p < 0.05, compared with the control group; **p < 0.01, compared with the control group.

Since the mTOR plays a key role in driving autophagy, we determined the expression of phosphorylated mTOR (Ser2448) and total mTOR in the spleen. When compared to the control group, the mRNA expression levels of mTOR were markedly decreased (P<0.05 or P<0.01) in the 24 and 48 mg/kg groups (Figure 10A). Furthermore, the protein expression levels of Ser2448 p-mTOR and total mTOR were significantly reduced (P<0.05 or P<0.01) in the 24 and 48 mg/kg groups (Figure 10B-10D).

#### Changes of mRNA and protein expression levels of mTOR regulation-related genes in spleen

**Figure 11:**
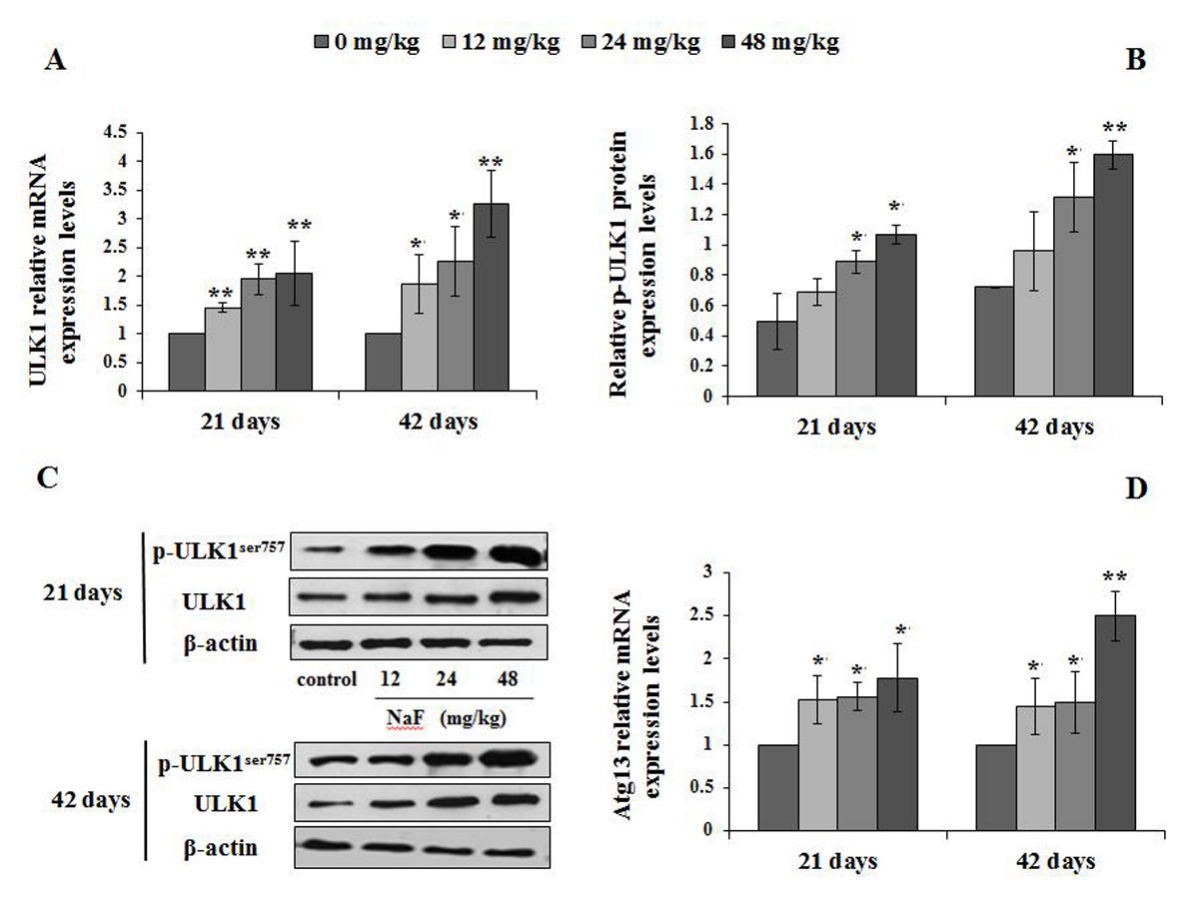
**Changes of ULK1 mRNA and protein expression levels and Atg13 mRNA levels in the spleen at 21 and 42 days of the experiment.** (**A**) The relative mRNA expression levels of ULK1. (**B**) The relative protein expression levels of ULK1. (**C**) The western blot assay of ULK1. (**D**) The relative mRNA expression levels of Atg13. Data are presented with the mean + standard deviation (n=8), *p < 0.05, compared with the control group; **p < 0.01, compared with the control group.

We then investigated the mTOR regulation-associated genes, including ULK1, PI3K, Akt and Atg13. The findings showed that ULK1 and Atg13 mRNA expression levels were significantly increased (P<0.01 or P<0.05) in the 12, 24 and 48 mg/kg groups at 21 and 42 days of the experiment (Figure 11A, 11D).

**Figure 12:**
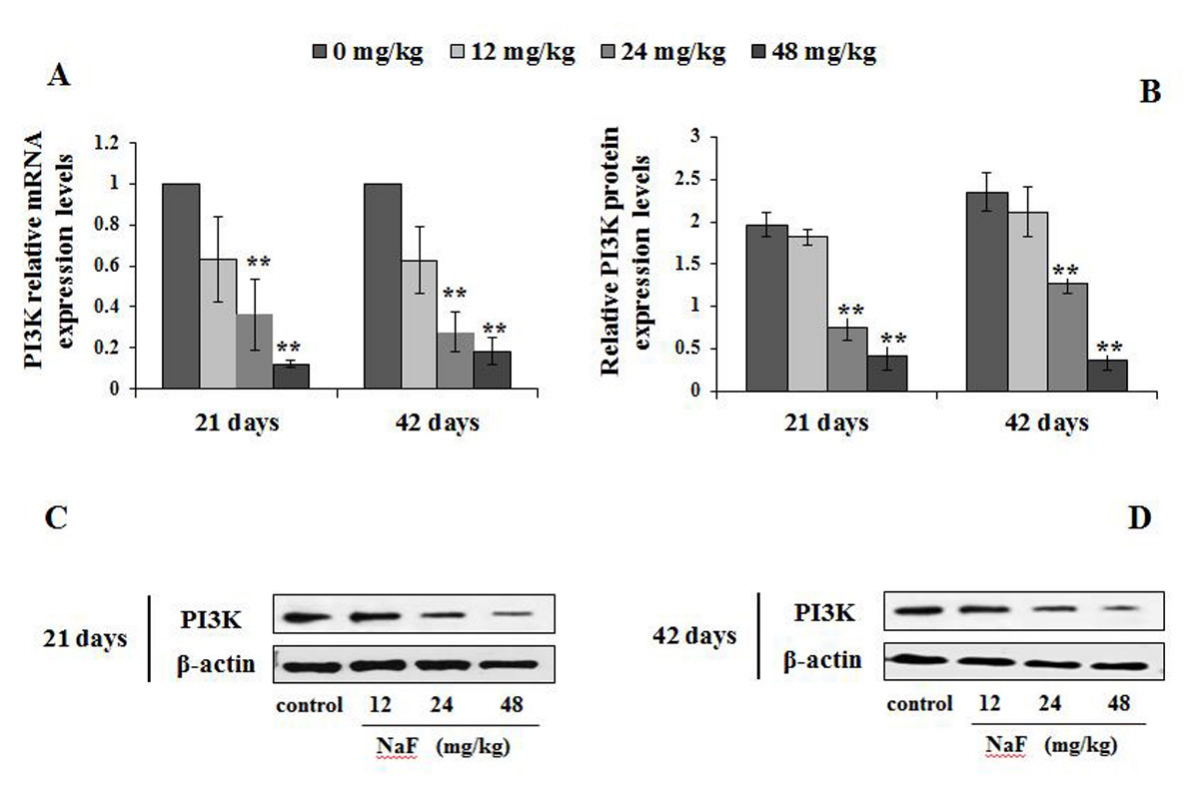
**Changes of mRNA and protein expression levels of PI3K in the spleen at 21 and 42 days of the experiment.** (**A**) The relative mRNA expression levels. (B) The relative protein expression levels. (C, D) The western blot assay. Data are presented with the mean + standard deviation (n=8), *p < 0.05, compared with the control group; **p < 0.01, compared with the control group.

**Figure 13:**
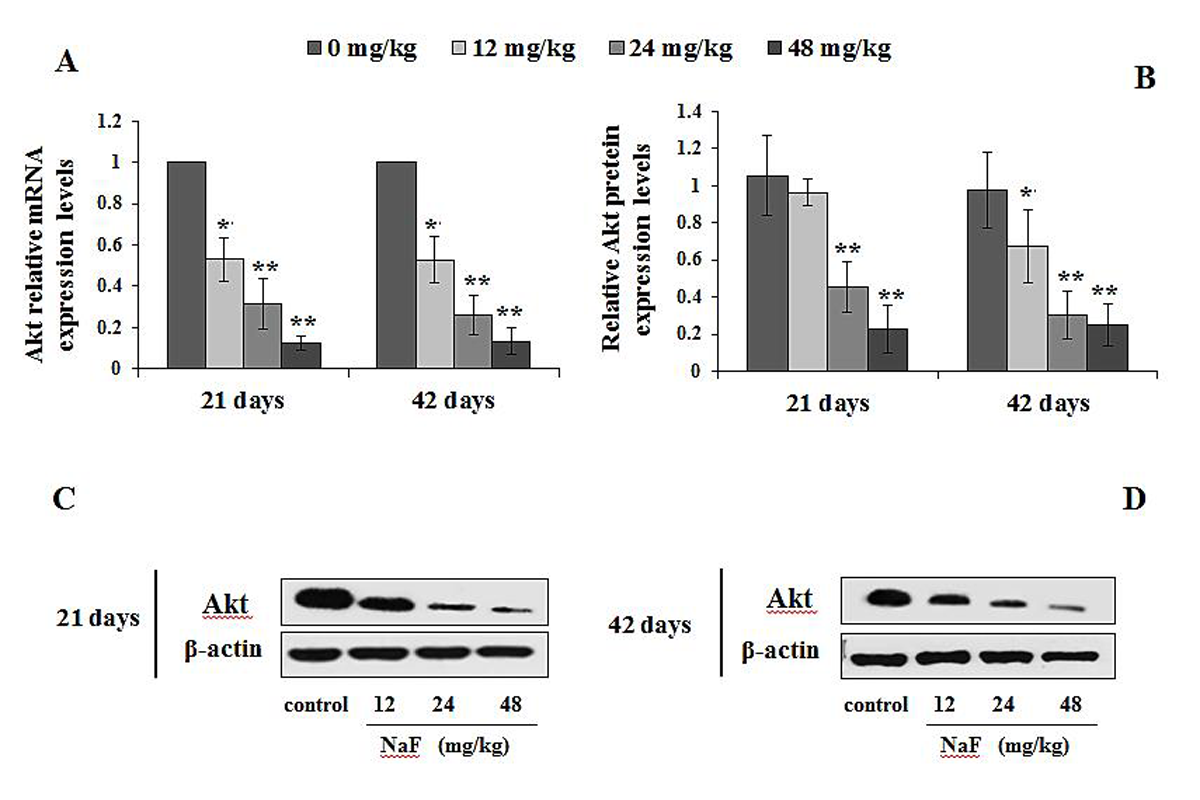
**Changes of mRNA and protein expression levels of Akt in the spleen at 21 and 42 days of the experiment.** (**A**) The relative mRNA expression levels. (**B**) The relative protein expression levels. (**C, D**) The western blot assay. Data are presented with the mean + standard deviation (n=8), *p < 0.05, compared with the control group; **p < 0.01, compared with the control group.

The p-ULK1 protein expression levels were significantly increased (P<0.05 or P<0.01) in the 24 and 48 mg/kg groups at 21 and 42 days of the experiment (Figure 11B-11C). At the same time, the PI3K mRNA and protein expression levels were significantly decreased (P<0.01) in the 24 and 48 mg/kg groups at 21 and 42 days of the experiment when compared to the control group (Figure 12). In addition, the Akt mRNA expression levels were significantly decreased (P<0.01 or P<0.05) in NaF-treated groups at 21 and 42 days of the experiment. Akt protein expression levels were significantly reduced (P<0.01or P<0.05) in 24 and 48 mg/kg groups at 21 days of the experiment and in 12, 24 and 48 mg/kg groups at 42 days of the experiment (Figure 13).

## DISCUSSION

Proteins are degraded via two main pathways in eukaryotic cells. Short-lived proteins are degraded by the proteasome, whereas long-lived proteins are degraded by autophagy [32]. In autophagy, cytoplasmic components are engulfed within a cytoplasmic vacuole by double-membrane-bound structures (autophagosomes) and delivered to lysosomes for degradation [33]. Thus, autophagy plays key roles in maintaining intracellular homeostasis by degrading and recycling damaged organelles and macromolecules [28]. It is presently unclear whether fluoride causes murine splenocyte autophagy, and the roles of autophagy in the splenic damage by fluoride treatment, which could contribute to elucidate the mechanism of fluoride-induced splenic toxicity in mice.

The appearance of autophagic vacuoles can be confirmed the occurrence of autophagy [20]. Once the autophagosome is formed, it must deliver its cargo to the lysosome in mammals or the functionally related vacuole in yeast and plants [34]. Then the outer membrane of autophagosome will fuse with lysosomal/vacuolar membrane to form the autolysosome [35]. Subsequently, the autophagic cargo are degraded and the component parts are exported back into the cytoplasm through lysosomal permeases for use by the cell in biosynthetic process or to generate energy [36]. We have observed that NaF caused an increase in the number of autophagosomes and autolysosomes in the mouse spleen (Figure1), which indicated that NaF induced autophagy in the spleen.

The LC3 serves as a autophagosomal marker protein in mammals. After being synthesized, LC3 is cleaved to produce the cytosolic LC3I (18 kDa) form. LC3I is converted to LC3II (16 kDa), which is tightly associated with the autophagosomal membrane via conjugation to phosphatidylethanolamine (PE) [37]. LC3II promotes autophagosome formation by facilitating membrane elongation [38]. It has been reported that alteration of LC3 expressions is involved in the fluoride-induced autophagy in mice leydig cells [28] or through ROS-mediated JNK signaling [39]. In addition, The punctate pattern of LC3 staining reflects the association of LC3-II with the membranes of early autophagosomes [40]. In the present study, the autophagy marker LC3 punctate staining was increased with NaF dosage increased (Figures 2-3), which showed that the increased punctate staining was corresponds to autophagosome buildup. Simultaneously, we noted that NaF significantly increased the LC3 mRNA and protein expression levels, and the ratio of LC3II/LC3I (Figure 4), which suggested that NaF likely caused a higher autophagic activity, and increased the formation of mature autophagosomes in the spleen. Additionally, LC3II expression levels were distinctly higher at 42 days of the experiment than 21 days of the experiment, which indicated that autophagy regulation was a dynamic process with time dependent pattern.

The p62 protein, also known as sequestosome-1 (SQSTM1) binding autophagy regulator Atg8/LC3 through a region termed the LC3-interacting region (LIR), suggests a link between autophagy and p62, whose levels can be regulated by autophagy [41]. When autophagy is inhibited, p62 accumulates, and when autophagy is induced, p62 is decreased [42]. Mathew et al. has verified that defective autophagy is a mechanism for p62 up-regulation commonly observed in human tumors, and contributes directly to tumorigenesis [43]. Similarly, Jaakkola et al. has reported that p62 down-regulated by hypoxia-activated autophagy in carcinoma cells [44]. Beclin1, as a protein involved in the initiation and execution of autophagy, regulates the autophagosome-lysosome fusion by interacting with Atg12-Atg5 and LC3-PE complexes [45]. Lei et al. has also demonstrated that high fluoride cause autophagy of HAT-7 cells by observing the expression of Beclin1 and mTOR to elucidate the mechanism of dental fluorosis [46]. Our study showed that the mRNA and protein expression levels of p62 were significantly decreased, while the Beclin1 levels were significantly enhanced in NaF-treated groups (Figures 5-6), which were consistent with the results of M Komatsu et al. [47] and Pattingre et al. [48] that p62 was regulated in controlling intracellular inclusion body formation by autophagy, and Beclin1 was involved in the initial step of autophagosome formation.

Atg5 was originally characterized as a binding partner with Atg12 in yeast, forming a complex that regulates the processing of LC3 and autophagosome formation [20]. Two ubiquitin-like (Atg12 and Atg8/LC3) conjugation systems in the downstream of Beclin-1- phosphatidylinoside 3- kinase are important for the induction of autophagy. The terminal product of one of two pathways is Atg5-Atg12 covalent complex, which is required for the elongation of the isolation membrane [49]. Atg16L1 forms an essential autophagy complex with Atg5 and Atg12 that facilitates elongation of the initial isolation membrane that results in engulfment of the cargo and formation of the autophagosome. Subsequent fusion with the lysosome facilitates degradation and allows nutrient recycling [50]. From this study, we found that the mRNA and protein expression levels of Atg12, Atg5 and Atg16L1 were markedly up-regulation (Figures 7-9), which indicated that splenocytes were sensitive to autophagy. Also, alteration of the above-mentioned LC3, p62, Beclin1, Atg12, Atg5 and Atg16L1 showed NaF-induced autophagy in this study.

**Figure 14:**
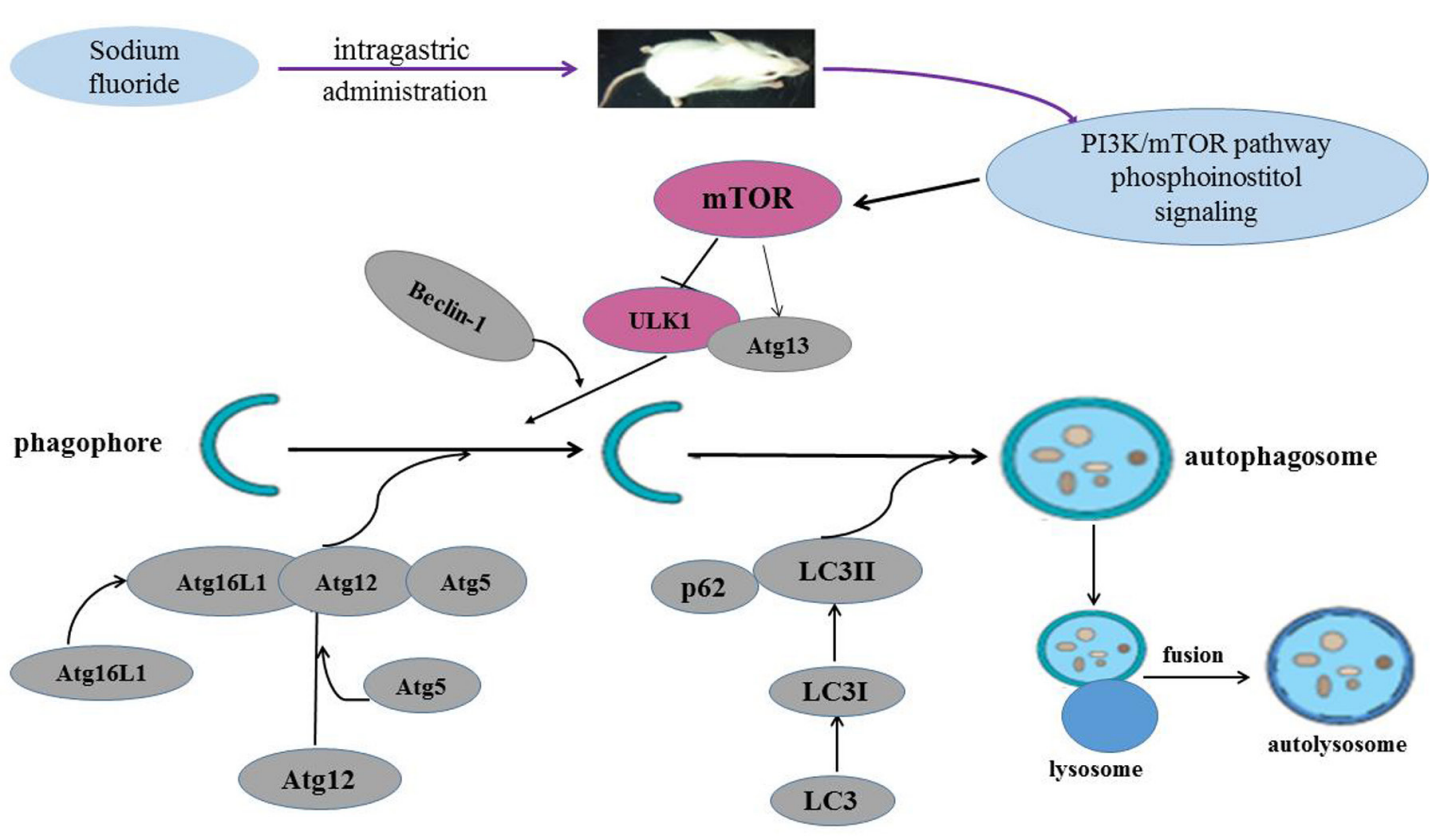
**Sodium fluoride induces autophagy in mammalian mice spleen.** NaF can induce the splenocyte autophagy by inhibiting the PI3K and mTOR activity, which in turn enhanced ULK1 and Atg13 expression levels, and then increased LC3, Beclin1, Atg16L1, Atg12, Atg5 expression levels, and reduced p62 expression level.

The regulatory role of the mTOR signaling pathway in autophagy was first demonstrated in yeast [51], and later in Drosophila [52]. As a central checkpoint that negatively regulates autophagy, the inhibition of mTOR activity initiates autophagy, and leads to dephosphorylation of ULK1 and Atg13 which relieved the inhibition of autophagy [53,54]. In this study, mTOR signaling pathway was suppressed (Figure 10), and the expressions of ULK1 and Atg13 were up-regulated (Figure 11) by NaF treatment. Autophagy is a cellular stress response that is activated through a number of pathways, mTOR integrates signals that either inhibit autophagy via the PI3K/Akt pathway [55] or trigger autophagy via activation of AMPK (Adenosine 5‘-monophosphate (AMP)-activated protein kinase) [56], and the activation of Akt (protein kinase B, PKB) can be regulated by PI3K [57]. Our study showed that PI3K and Akt expression levels were significantly declined (Figures 12-13) in NaF-treated groups, which indicated that NaF led to the suppression of mTOR signaling and promoted autophagy. The relationship between autophagy and mTOR regulation-related genes is shown in Figure 14.

Numerous autophagy-related genes and proteins participate in the initiation and formation of autophagosomes and in cargo recognition [45]. The complete autophagosome is then transported to fuse with lysosomes, and degradation of the content releases valuable anabolic compounds. The present study finds that NaF in excess of 12 mg/kg can induce the splenocyte autophagy via inhibition of mTOR activity, which is characterized by down-regulation of PI3K/Akt expression and p-mTOR expression. And the suppression of mTOR activity in turn initiates autophagy by up-regulating ULK1 and Atg13 expression. Increased or decreased expression levels of the LC3II, p62, Beclin1, Atg16L1, Atg12 and Atg5 promote the initiation and formation of autophagosome. The inhibition of mTOR activity and alteration of autophagy-related genes and proteins are the potential molecular mechanism of NaF-induced splenocyte autophagy. The above-mentioned results may provide new insights for further understanding the role of autophagy in fluoride-induced splenic damage and toxicity.

## MATERIALS AND METHODS

### Animals and treatment

240 healthy ICR mice (Experimental Animal Corporation of DOSSY at Chengdu, China) were used in this study to estimate NaF-induced autophagy in the spleen. Food and water was provided *ad libitum*. Mice were randomly divided into 4 groups (*N* = 60). The control group was given an intragastric administration of distilled water at the same time as other groups. The experimental groups were given an intragastric administration of 12, 24, and 48 mg/kg NaF (Chengdu Kelong Chemical Co., Ltd., Chengdu, China), respectively. The gavage doses of four groups were 1 mL/100 g body weight once daily for the last 42 days.

Our experiments involving the use of mice and all experimental procedures were approved by the Animal Care and Use Committee, Sichuan Agricultural University.

### Determination of autophagosomes and autolysosome in the spleen by transmission electron microscopy (TEM)

The spleen were rapidly cut into ∼1 mm × ∼1 mm × ∼1 mm pieces, fixed in 2.5% glutaraldehyde at room temperature. After fixation, a 0.2M phosphate buffer (pH 7.2) was used to rinse the tissue twice for 15 minutes. Next 1% buffered osmium tetroxide was used to post-fix the samples for 1 h, dehydrated in a graded series of ethyl alcohol, and embedded in epoxy resins. The ultrathin sections were prepared, mounted on copper grids, and stained with uranyl acetate and lead acetate. Then, the images were examined and photographed using transmission electron microscope.

### Determination of autophagy marker in the spleen by immunohistochemistry (IHC)

Spleen was fixed in 4% paraformaldehyde overnight followed by dehydration in ethanol, embedded in paraffin wax.

The spleen paraffin sections were dewaxed in xylene, rehydrated through a graded series of ethanol solutions, washed in distilled water and PBS and endogenous peroxidase activity was blocked by incubation with 3% H_2_O_2_ in methanol for 15 min. The slices were subjected to antigen retrieval procedure by microwaving in 0.01 M sodium citrate buffer pH 6.0. Additional washing in PBS was performed before 30 min of incubation at 37 °C in 10% normal goat serum (Boster, Wuhang, China). Then incubated overnight at 4 °C with the primary antibodies (1:100). After washing in PBS, the slices were exposed to 1% biotinylated goat anti-rabbit IgG secondary antibody (Boster, Wuhang, China) for 1 h at 37°C, and then incubated with strept avidinbiotin complex (SABC; Boster, Wuhang, China) for 30 min at 37 °C. To visualize the immunoreaction, slices were immersed in diaminobenzidine hydrochloride (DAB; Boster, Wuhang, China). The slices were monitored microscopically and stopped by immersion in distilled water, as soon as brown staining was visible. Slices were lightly counterstained with hematoxylin, dehydrated in ethanol, cleared in xylene and mounted. For negative control purposes, representative sections were processed in the same way by replacing primary antibodies by PBS.

### Determination of mTOR signaling pathway parameters’ mRNA expression levels in the spleen by qRT-PCR

At 21 and 42 days of the experiment, spleens of eight mice in each group were removed and stored in liquid nitrogen, and then homogenized with liquid nitrogen by a mortar and pestle, and Total RNA was extracted with the RNAiso Plus (9108/9109, Takara, Japan) according to manufacturer’s instructions. Then, cDNA was synthesized with the Prim-Script™ RT reagent Kit (RR047A, Takara, Japan) according to the manufacturer’s instructions and used as a template for qRT-PCR. Specific primers for the genes were designed with the Primer 5 software and synthesized by Sangon (Shanghai, China) (Table 1).

**Table 1:** Sequence of primers used in qRT-PCR.

**Gene symbol**	**Accession number**	**Primer**	**Primer sequence(5'-3')**	**Product size**	**Tm(°C)**
mTOR	NM-020009.2	Forward	CAGACTGGCTCTTGCTCATAA	155 bp	57
		Reverse	GCTGGAAGGCGTCAATC		
ULK1	NM-009469.3	Forward	ACACACCTTCTCCCCAAGTG	198 bp	60
		Reverse	GACGCACAACATGGAAGTCG		
Beclin1	NM-019584.3	Forward	TGCAGGTGAGCTTCGTGTG	124 bp	60
		Reverse	GCTCCTCTCCTGAGTTAGCCT		
Atg16L1	NM-001205392.1	Forward	CTGAGAAGGCCCAAGAAGCC	221 bp	60
		Reverse	GACAGAGCGTCTCGTAGCTG		
Atg12	NM-026217.3	Forward	TAAACTGGTGGCCTCGGAAC	146 bp	60
		Reverse	ATCCCCATGCCTGGGATTTG		
Atg5	NM-053069.6	Forward	CAAGGATGCGGTTGAGGC	167 bp	58
		Reverse	TGAGTTTCCGGTTGATGG		
LC3	NM-025735.3	Forward	CTTCGCCGACCGCTGTAA	170 bp	60
		Reverse	GCCGGATGATCTTGACCAACT		
Atg13	NM-145528.3	Forward	ACTGGTGATGCACATGCCTT	149 bp	50
		Reverse	ATGCTCCCACTTTTCGGACA		
p62	NM-011018.3	Forward	GCACAGGCACAGAAGACAAG	134 bp	59
		Reverse	CACCGACTCCAAGGCTATCT		
PI3K	NM-001077495.2	Forward	CTGGGGGACATACTGACTGT	140 bp	60
		Reverse	GTTCCTGGAAAGTCTCCCCTC		
Akt	NM-009652.3	Forward	TCCTCAAGAACGATGGCACC	203 bp	60
		Reverse	CTCCTCAGGCGTTTCCACAT		
β-actin	NM-007393	Forward	GCTGTGCTATGTTGCTCTAG	117 bp	60
		Reverse	CGCTCGTTGCCAATAGTG		

qRT-PCR was carried out in a LightCycler 96 (Roche, Germany) using SYBR® Premix Ex Taq^TM^ II (DRR820A, Takara, Japan) according to the standard protocols. The melting curve analysis was performed to ensure a single peak for each PCR product. Further, purity of specific PCR products was verified by agarose gel electrophoresis. Mouse β-actin was used as an internal reference. Gene expression at days 21 and 42 were calibrated against the corresponding controls. Relative expression was analyzed by the 2^-ΔΔCT^ method [58].

### Determination of mTOR signaling pathway parameters’ protein expression levels in the spleen by Western blot

At 21 and 42 days of experiment, splenic samples of eight mice in each group were taken to determine the autophagy protein expression levels by western blot.

The proteins were extracted from frozen spleen samples with RIPA lysis buffer (P0013C; Beyotime, China) and quantified by the BCA Protein Assay Kit (P0012; Beyotime, China). Equal amounts of protein samples were resolved on SDS-PAGE (10%–15% gels) and transferred to nitrocellulose filter membranes. Membranes were blocked with 5% fat-free milk for 1h and incubated with primary antibodies overnight at 4°C. The primary antibodies were mTOR, p62, PI3K, Akt (Abcam, UK), ULK1, Beclin-1, Atg16L1, Atg12, Atg5, LC3 (CST, USA). The membranes were then washed with PBS-tween and incubated with biotin-conjugated secondary antibodies (CST, USA) for 1h, and washed again with PBS-tween. Blots were visualized by ECL^TM^ (Bio-Rad, Hercules, CA, USA) and X-ray film. The protein bands were quantified with the Image J software.

### Statistical analysis

The experimental data are expressed as the mean ± standard deviation. One-way analysis of variance (ANOVA) procedure in SPSS 19.0 software was used to assess statistical significances between F-treated group and control group. A value of *P* < 0.05 or *P* < 0.01 was accepted as significant differences.
